# Synergistic association of FOXP3^+^ tumor infiltrating lymphocytes with CCL20 expressions with poor prognosis of primary breast cancer: A retrospective cohort study

**DOI:** 10.1097/MD.0000000000018403

**Published:** 2019-12-16

**Authors:** Xia Zhao, Yanping Li, Xiaoli Wang, Jiangping Wu, Yanhua Yuan, Shuzhen Lv, Jun Ren

**Affiliations:** a Department of Medical Oncology, Beijing Key Laboratory for Therapeutic Cancer Vaccines; b Department of Surgical Breast Cancer, Capital Medical University Cancer Center, Beijing Shijitan Hospital, Capital Medical University, Beijing, China; c Department of Surgery, Duke University Medical Center, Durham, NC, US.

**Keywords:** breast cancer, CCL20, disease-free survival, FOXP3^+^ tumor infiltrating lymphocyte, overall survival, synergistic association

## Abstract

Studies have shown that forkhead/winged helix transcription factor P3 (FOXP3)^+^ tumor infiltrating lymphocytes (TILs) are intimately associated with invasion and survival of many invasive tumors. The inflammatory chemokine ligand 20 (CCL20) and its receptor CCR6 were found to be associated with tumor prognosis in some studies. Although increases in FOXP3^+^ TILs infiltration and CCL20 expression have been revealed in several malignancies, their correlation in human breast tumors is as yet unclear.

Surgically resected samples from 156 patients with invasive breast cancer (BC) were assessed for the expression of FOXP3 and CCL20 by immunohistochemistry. Correlation between their expressions and the association with clinicopathological characteristics and patient's prognosis were studied. Forty pairs of fresh BC and their nontumor adjacent tissues (NATs) in BC were carried out by real-time quantitative PCR (qRT-PCR) to evaluate the correlation between FOXP3 and CCL20 mRNA expression.

CCL20 and FOXP3^+^ TILs mRNA expression in tumor tissue demonstrated a high correlation (rs = 0.359, *P* < .001) in this cohort of breast cancer patients. Both elevated CCL20 expression and FOXP3^+^ TILs infiltration were significantly correlated with high histological grade, positive human epidermal growth factor receptor-2 (HER2), high Ki67 index, and axillary lymph node metastases. Tumors with concomitant high expressions of both markers had the worst prognosis. Multivariate analysis showed that these 2 markers were independent predictors of overall survival. The patients with axillary lymph node metastases with the concomitant CCL20 high expression and increased FOXP3^+^ TILs infiltration had the worst overall survival (OS) (*P* < .001), In lymph node-negative breast cancer patients, the status of CCL20 and FOXP3 was not related to OS (*P* *=* .22).

The results suggest that CCL20 and FOXP3^+^ TILs may have synergistic effects, and their upregulated expressions may lead to immune evasion in breast cancer. Combinatorial immunotherapeutic approaches aiming at blocking CCL20 and depleting FOXP3 might improve therapeutic efficacy in breast cancer patients.

## 1 Introduction

According to statistics from the International Agency for Research on Cancer GLOBOCAN database, the global incidence of breast cancer is ranked first among cancers in women. In 2015, the United States had approximately 231,840 new cases of invasive breast cancer and 40,290 cases of breast cancer-related deaths.^[1]^ In China, breast cancer shows an overall increasing trend in the last few decades, an annual increase of 3% to 5% and a 5-year overall survival rate of 72.7%.^[2]^ Relapse and metastasis are the primary causes of death in breast cancer patients. Around 30% to 40% of breast cancer patients will progress to metastatic disease, with a median survival of 3 years.^[2]^

Effective immune evasion by cancer cells is a crucial step during cancer occurrence, progression, and metastasis. As functional immunosuppressive T-cells, regulatory T-cells (Tregs) play important roles in immune tolerance and immune evasion.^[3,4]^ Currently, the inhibitory mechanisms of Tregs are still unclear. However, more and more evidence has shown that increased numbers of Tregs in tumor-infiltrating lymphocytes (TILs) or peripheral blood mononuclear cell (PBMCs) may be one of the reasons why the host has impaired tumor immunity.^[5,6]^ Tregs can directly contact cells or secrete immunosuppressive cytokines to indirectly inhibit the functions of effector T-cells.^[7,8]^ FOXP3 predominantly expressed within the nuclear functions as a type of forkhead box transcription factor containing a DNA-binding domain. It can simultaneously recruit transcriptional activator and repressor protein complexes.^[9]^ As a specific biomarker, FOXP3 is used to identify Tregs in inflammatory infiltrates. FOXP3 also plays an important role in the development and function of Tregs.^[10]^

The tumor microenvironment is rich in molecules and several possible mechanisms may increase the number of FOXP3^+^ Tregs, such as driving CD4^+^ T-helper cells to develop into FOXP3^+^ Tregs, recruiting existing FOXP3^+^ Tregs to tumor sites, and inducing the expansion of retained Tregs. This form of tumor-induced FOXP3^+^ Treg elevation represents a potential obstacle for cancer immunotherapy.^[11,12]^

There were some studies to demonstrate the prognostic significance of FOXP3^+^ Tregs which could lead to poor outcomes in many cancers, such as prostate cancer, lung cancer, hepatocellular carcinoma, and renal cell carcinoma.^[13–17]^

In breast cancer, the prognostic significance of FOXP3^+^ Tregs is widely varied. Recent studies have reported that infiltration of FOXP3^+^ Tregs is associated with poor clinical outcomes in breast cancer^[18–22]^ but a cohort study on 175 patients with estrogen receptor (ER) negative breast cancer found that the FOXP3^+^ TILs and CD8^+^ TILs showed a positive correlation and that this relationship was an independent good prognostic factor for ER-negative breast cancer.^[23]^ A study of triple-negative breast cancer showed FOXP3^+^ Tregs to be a significant and independent factor associated with better overall survival and progression-free survival.^[24]^ The characteristics of FOXP3^+^ TILs may be affected by the tumor microenvironment. Therefore, the prognostic value of FOXP3^+^ TILs may be affected by breast cancer molecular subtypes and interactions with other immune cells.

The tumor microenvironment plays a significant role in cancer maintenance and progression, as well as the recruitment of inflammatory cells to tumor sites; the resulting microenvironment facilitates cancer progression. Chemokines are small (8–14 kDa) secreted proteins that play key roles in recruiting and modulating the activity of inflammatory cells by interacting with their corresponding receptors.^[25]^ Chemokine ligand 20 (CCL20), the ligand for CCR6, is secreted by tumor and stromal cells where they recruit macrophages, and leukocytes such as TILs to the tumor location.^[26]^ Studies have shown CCL20 and CCR6 to be significantly upregulated in several solid tumors and closely associated with poor prognosis.^[27–30]^

Here, we have used immunohistochemistry and qRT-PCR assays to assess the correlation between CCL20 and FOXP3^+^ TIL expressions and the association with clinicopathological characteristics, analyzed the prognostic significance of CCL20 expression and FOXP3^+^ TILs infiltration in breast cancer, and deduced that CCL20/CCR6 may recruit FOXP3^+^ TILs to tumor sites, thereby promoting immune evasion in tumors.

## 2 Materials and methods

### 2.1 Patients and samples

In this retrospective study, we had collected 156 samples from patients with invasive breast cancer from January 2009 to May 2013. These patients all underwent radical breast cancer surgery at Beijing Shijitan Hospital, Capital Medical University. The primary diagnosis of every patient was determined by HE staining. Among these patients, 139 had invasive ductal carcinoma (IDC), 13 had invasive lobular carcinoma (ILC), and 5 had cancers of other histological types. Immunohistochemical (IHC) staining was used to assess the expression of estrogen receptor (ER), progesterone receptor (PR), human epidermal growth factor receptor-2 (HER2), and Ki67. The breast cancer subtype was defined by the IHC expression of ER, PR, HER2, and Ki67 (according to the 2007 American Society of Clinical Oncology/College of American Pathologists (AS4CO/CAP) guidelines).^[31]^ Tumor staging was carried out according to the 7th edition of the AJCC TNM classification.^[32]^

All patients were female and the mean age at breast cancer diagnosis was 51 years (32–70 years). The follow-up period was 32 to 72 months and the median follow-up period was 51 months. All patients had tumors that were localized to the breast and there was no evidence of distal metastases or skin involvement. All cancers were removed by surgical resection and 74 patients underwent axillary lymph node dissection. No patient underwent neoadjuvant chemotherapy or preoperative radiotherapy. After surgery, 138 patients underwent adjuvant chemotherapy with paclitaxel + cyclophosphamide or paclitaxel + epirubicin, 60 patients underwent radiotherapy, and 114 patients received endocrine therapy.

In addition, we collected 40 pairs of fresh tumor tissues and their nontumor adjacent tissues (NATs) (5 cm from the tumors) from January to October 2017 from patients who underwent radical breast cancer surgery. Samples were collected during surgery and immediately placed in liquid nitrogen tanks. After surgery, the samples were transferred to −80°C freezer for storage. The preoperative pathological diagnoses of all these samples were IDC, and patients did not receive neoadjuvant chemotherapy or preoperative radiotherapy.

All patients signed the informed consent before surgery, authorized the use of surgical samples in this clinical study. This study was also approved by the research ethics committee of Beijing Shijitan Hospital.

### 2.2 Immunohistochemical staining and evaluation

We have collected tissue samples from 156 patients. These were stored in the Department of Pathology in Beijing Shijitan Hospital, Capital Medical University from January 2009 to May 2013. These samples were all from tissue blocks that were fixed in formalin after surgery and embedded in paraffin. The paraffin-embedded tissues were cut into 3 μm thickness sections, followed by xylene clearing, PBS washing, and high-pressure repair: The repair solution (pH 8.0) was preheated in an autoclave before 3% hydrogen peroxide was added and incubated at room temperature for 10 to 15 min. In accordance with the manufacturer's instructions, the sections were incubated with mouse anti-FOXP3 monoclonal antibodies (Abcam, Cambridge, UK ab20034, clone 236A/E7) for 30 minutes at room temperature in a dilution of 1:100. For CCL20 and CD4, each section was incubated with rabbit polyclonal anti-human CCL20 (1:50 dilution, ab9829, Abcam, Cambridge, UK) and anti-CD4 (1:200 dilution, ab133616, Abcam, Cambridge, UK) in the dark at 4°C overnight. Secondary antibodies and peroxidase-conjugated streptavidin were purchased from DAKO. The substrate was 4,3-diaminobenzidine tetrahydrochloride. Phosphate-buffered saline was used to incubate sections for negative control.

The immunohistochemical expression of all the samples was independently evaluated by 2 pathologists who will assess the expression of the antibodies in the cell membrane, cytoplasm, or nucleus. The pathologists are both unaware of the clinicopathological data. Both the pathologists carried out a joint review of differences and achieved a consensus.

Among the 156 samples, the median percentage of FOXP3 expression was 10%, the expression of 105 samples was 0% to 10%, which was determined to be low expression; the expression of 51 samples was 10% to 90%, which was determined to be high expression. The median percentage of CD4 expression was 80%. The expression in 131 samples was 30% to 79%, which was here considered relatively low; the expression in the other 25 samples was 80% to 95%, which was here considered high expression. The immunohistochemical staining CCL20 was determined by the proportion of positive cells and staining intensity. The results of immunostaining were determined by the number of positive cells and staining intensity (the percentage of cells stained: 0=0, 1=1–10%, 2=11–50%, 3=51–80%, 4=81–100%; and staining intensity: negative =0, weak =1, moderate =2, strong =3). The intensity of the immunostaining score and the percentage-of immunoreactive cells score were multiplied to generate the immunoreactive score (IRS). The IRS ranged from 0 to 12, with ≤4 being regarded as low expression and >4 being regarded as high expression. Immunohistochemical scores were independently determined by 2 pathologists without access to patient characteristics.

### 2.3 Quantitative real-time PCR

RNA was extracted from 40 pairs of fresh surgical samples (fresh tumor tissues and their NATs that were stored in liquid nitrogen) according to the manufacturer's instructions (Invitrogen, USA). A high-capacity RNA-to-cDNA Kit (Applied Biosystems 4387406) was used to reverse transcribe 2 μg RNA into cDNA according to the manufacturer's instructions. qRT-PCR of the samples using FOXP3, CCL20, and glyceraldehyde-3-phosphate dehydrogenase (GAPDH) (housekeeping gene) primers was carried out on an AB7500 qRT-PCR machine. The primers used were (5′–3′): CCL20: forward, ATTGTGCGTCTCCTCAGTAAAAA and reverse, TGTGATGCTTAAACAAAGCAAAC and FOXP3: forward, TCCAGGACAGGCCACATTTC and reverse, GGGATTTGGGAAGGTGCAGA. The data were measured and normalized to GAPDH expression as a reference.

### 2.4 Follow-up

The follow-up period was defined as the period from the day of breast cancer surgery to death or last follow-up. Overall survival (OS) was defined as the period from the day of breast cancer surgery to death from any cause or last follow-up. Disease-free survival (DFS) was defined as the period from the day of breast cancer surgery to any recurrence or metastasis of the disease. The follow-up cycle was once every 3 months in the first 2 years, followed by once every 6 months for 3 years and above.

### 2.5 Statistical analysis

The IBM SPSS statistical software version 21 (SPSS Inc, Chicago, IL) was used for statistical analysis. The Spearman rank correlation test was used to evaluate the relationship between FOXP3^+^ TILs infiltration; CCL20, and clinicopathological characteristics. Pearson correlation test was used on continuous data to determine the strength of the correlation between FOXP3 and CCL20 mRNA expression levels. The Kaplan–Meier method was used to plot survival curves and the log-rank test was used for analysis. The Kaplan–Meier method was used to plot survival curves and the log-rank test was used to evaluate the significance of OS differences between the subgroups. A Cox proportional hazard regression model was constructed for univariate and multivariate analyses. All tests were 2-tailed and a *P* value < .05 was considered statistically significant.

## 3 Results

### 3.1 General status

The median age of the 156 breast cancer patients was 51 years. There were 88 patients ≤50 years old and 68 patients >50 years old. There were 114 patients who were ER-positive, 45 patients who were HER-2-positive, and 28 patients with triple-negative breast cancer. There were 74 patients with axillary lymph node metastases, of whom 33 patients had ≥4 lymph node metastases. In the 156 breast cancer patients, the follow-up period ranged from 32 to 72 months and median survival was 51 months. There were 31 deaths and 44 patients with postoperative recurrence and distal metastases. Table 1 shows the clinicopathological characteristics of these patients.

**Table 1:**
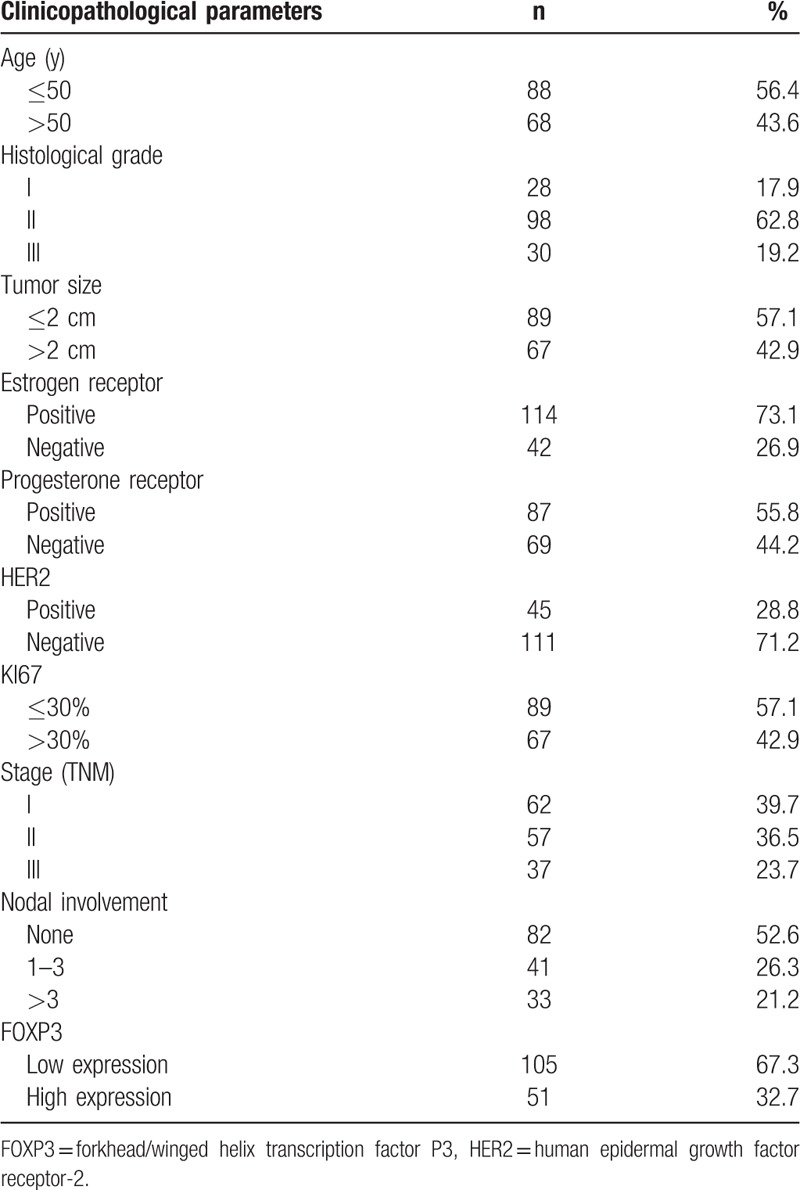
Clinicopathologic characteristics of breast cancer patients (n = 156).

### 3.2 Correlation of CCL20 expression with FOXP3^+^ TILs infiltration and clinicopathological features

**Figure 1:**
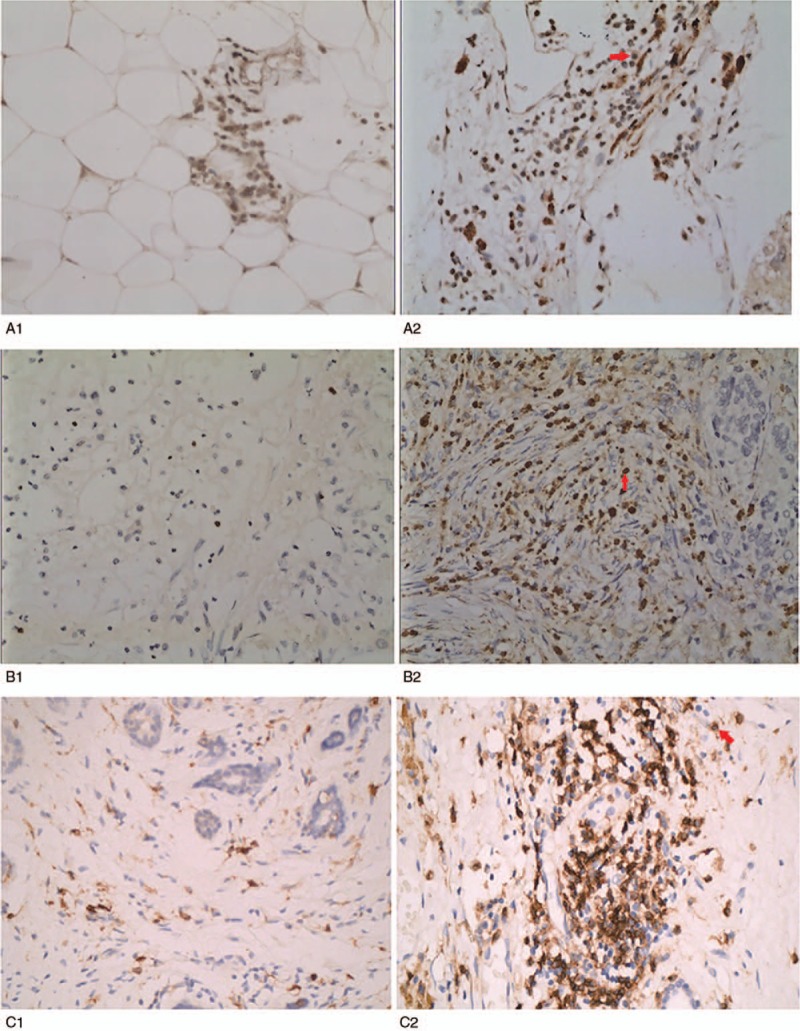
Immunohistochemical staining using primary antibodies against CCL20, FOXP3, CD4. Original magnification, 400×, the red arrow to mark the staining site. A, Representative immunohistochemistry staining for CCL20 in BC. Frozen sections of BC tissue specimens were prepared for immunohistochemical staining using rabbit polyclonal antihuman CCL20 (1:50 dilution, ab9829; Abcam, Cambridge, UK). (A1) CCL20 low expression (A2) CCL20 high expression. B, The infiltrating density of intratumoral FOXP3^+^ TILs in BC was detected using mouse monoclone anti-FOXP3 (clone A236A/E7), the location of FOXP3 expression was within the cell nucleus. (B1) FOXP3 low expression (B2) FOXP3 high expression. C, The infiltrating density of intratumoral CD4^+^ TILs in BC was detected using anti-CD4 antibody (dilution 1:200, ab133616, Abcam), CD4 were clearly stained in the cell membranes. (C1) CD4 low expression, (C2) CD4 high expression. BC = breast cancer, CCL20 = chemokine ligand 20, FOXP3 = forkhead/winged helix transcription factor P3, TIL = tumor infiltrating lymphocyte.

We used immunohistochemistry to quantitate FOXP3, CD4, and CCL20 expression in 156 invasive breast cancers samples (Fig. 1). Of these samples, 51 cases (32.7%) had high FOXP3^+^ TIL infiltration, 25 cases (16.0%) had high levels of CD4^+^ expression, and 92 (59.0%) cases exhibited high CCL20 expression. We have observed that the location of FOXP3 expression was within the cell nucleus as similar with other previously published.^[33]^ with contrast to only a few samples (n = 5) showing simultaneous cytoplasm staining (Table 2). Scores were calculated by observing the areas of expression in the nucleus. CD4 were clearly stained in the cell membranes of tumor-infiltrating cells. The percentage was calculated in the areas showing the highest expression of FOXP3 or CD4. In the 156 breast cancer patients, CCL20 expression was significantly correlated with high histological grade (*P* = .008), positive lymph node metastases (*P* = .02), positive HER2 (*P* = .02), and high Ki67 index (*P* = 0.03). FOXP3 expression was positively correlated with high histological grade (*P* = .04), positive lymph node metastases (*P* = .01), negative PR (*P* = .03), positive HER2 (*P* = .02), and high Ki67 index (*P* = .005). High CCL20 expression and increased FOXP3^+^ TILs infiltrates were both associated with high histological grade, axillary lymph node metastases, positive HER2, and high Ki67 index but not correlated with age, tumor size, ER status. CD4 expression was not found to be significantly correlated with the clinicopathological characteristics of all patients. However, in patients with high levels of FOXP3 expression (n = 51), CD4 expression was significantly positively correlated with positive HER2 (*P* = .001). In addition, a significant correlation was observed between CCL20 expression and FOXP3^+^ TILs infiltration in breast cancer tissue (rs = 0.359, *P* < .001) (Table 2).

**Table 2:**
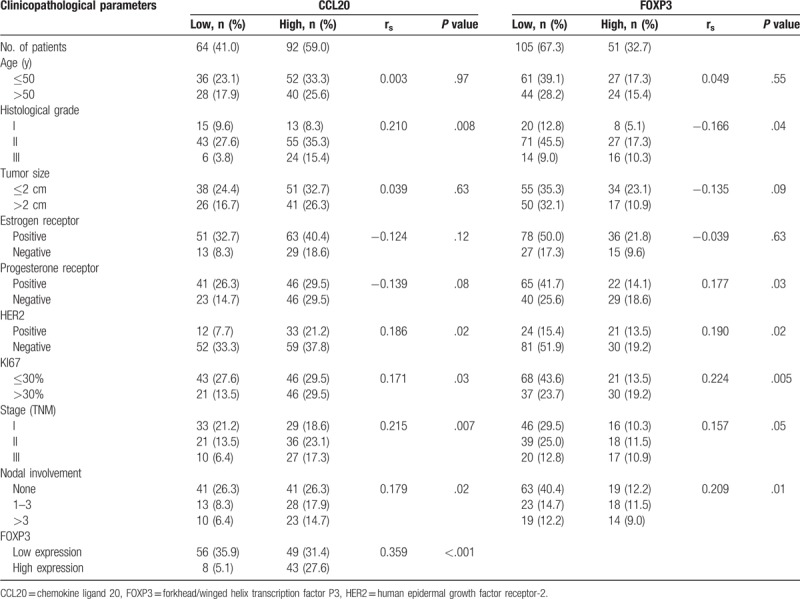
Associations between CCL20 expression and clinicopathological parameters (n = 156).

### 3.3 Prognostic significance of CCL20 expression and FOXP3^+^ TIL infiltration

**Figure 2:**
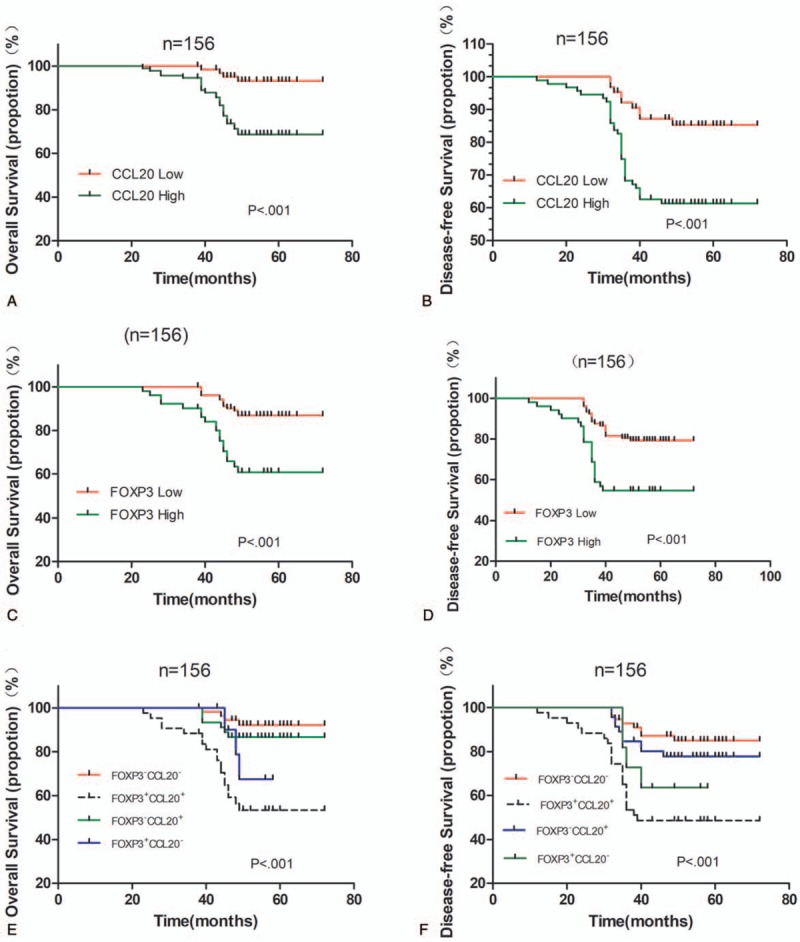
Kaplan–Meier survival curve for (A) OS and (B) DFS depending on the expression CCL20. (C) OS and (D) DFS depending on the tumor FOXP3^+^ TILs infiltration in breast cancer. (E) OS and (F) DFS depending on the CCL20 expression combined with the tumor FOXP3^+^ TILs infiltration. *P* values were calculated by the log-rank test. CCL20 = chemokine ligand 20, DFS = disease-free survival, FOXP3^+^CCL20^+^ = CCL20 high expression and increased FOXP3^+^ TILs infiltration, FOXP3^+^CCL20^−^ = CCL20 low expression and increased FOXP3^+^ TILs infiltration, FOXP3^−^CCL20^+^ = CCL20 high expression and decreased FOXP3^+^ TILs infiltration, FOXP3^−^CCL20^−^ = CCL20 low expression and decreased FOXP3^+^ TILs infiltration, FOXP3 = forkhead/winged helix transcription factor P3, OS = overall survival.

In order to determine the significance of the effects of clinicopathological factors on breast cancer survival, we carried out univariate analysis (Table 3). Results revealed that both CCL20 expression and infiltration of FOXP3^+^ TILs were unfavorable predictors for OS (*P* < .001; *P* < .001) and DFS (*P* = .001; *P* < .001) (Table 3). The high CCL20 expression group had significantly worse DFS and OS, than the low CCL20 expression group as determined by the Kaplan–Meier method and log-rank test (*P* < .001) (Fig. 2A, B). The 5-year DFS rate were 62.0% for the high CCL20 group and 85.9% for the low CCL20 group, the 5-year OS rate were 70.7% for the high CCL20 group and 93.8% for the low CCL20 group. The Kaplan–Meier survival analysis showed that FOXP3 expression in tumors is significantly correlated with the OS and DFS of all breast cancer patients (*P* < .001; *P* < .001, Fig. 2C, D). In addition, when the survival of patients with low FOXP3 expression was compared with patients with high FOXP3 expression, it was found that when FOXP3 staining intensity increased, the risk of survival also increased (*P* < .001) and patients with low FOXP3 expression had significantly greater DFS than patients with high FOXP3 expression. Tumor size (*P* = .02), histological grade (*P* = .01), ER status (*P* < .001), PR status (*P* < .001), HER-2 expression (*P* = .004), Ki67 index (*P* < .001) are also significantly correlated with OS but not age and CD4 expression. However, among all patients with high levels of FOXP3 expression, those in the high CD4 expression group had significantly worse OS than those in the low CD4 expression group (*P* = .05), the 5-year OS rates being 52.9% for the high CD4 group and 76.5% for the low CD4 group.

**Table 3:**
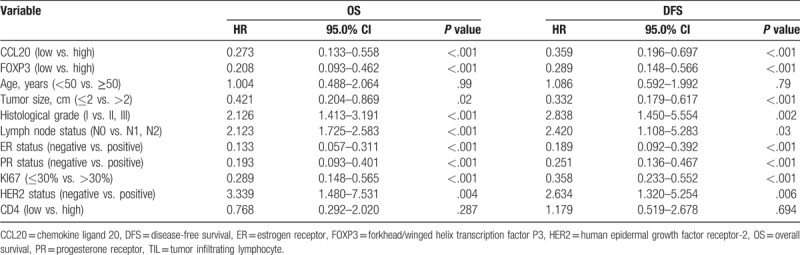
Univariate analysis of pathological features, CCL20 expression, and FOXP3^+^ TILs infiltration with OS and DFS in breast cancer patients (n = 156).

In order to assess the prognostic value of clinicopathological characteristics through the entire population of breast cancer patients, we constructed a Cox proportional hazards regression model to assess the hazards ratio of all parameters (age, tumor size, grade, ER status, PR status, HER-2 expression, lymph node metastases, Ki67 index, FOXP3 expression, and CCL20 expression) on breast-cancer-specific survival (Table 4). Both CCL20 expression and FOXP3^+^ TILs infiltration were independent prognostic factors for OS (HR = 3.389, *P* = .03; HR = 2.700, *P* = .02). FOXP3^+^ TILs infiltration was also independent prognostic factor for DFS (HR = 2.090, *P* = .03).

**Table 4:**
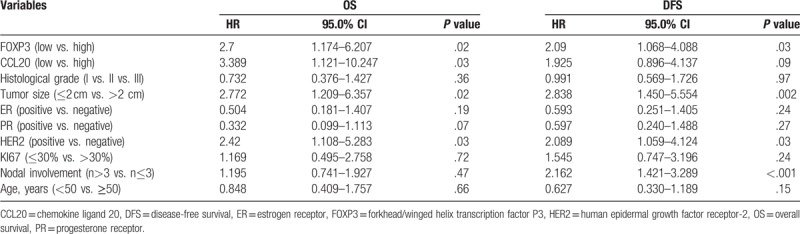
Multivariate analyses of variables associated with OS and DFS in breast cancer patients (n = 156).

### 3.4 Prognostic significance of concomitant CCL20 expression and FOXP3^+^ TILs

Forty-three (27.6%) tumors showed concurrence of CCL20 high expression and increased FOXP3^+^ TILs infiltration, while 56 (35.9%) tumors exhibited CCL20 low expression and decreased FOXP3^+^ TILs infiltration, and the other 57 (36.5%) tumors demonstrated neither of the above (Table 5). The group of the patients with the concomitant CCL20 high expression and increased FOXP3^+^ TILs infiltration showed the worst OS and DFS, while those with CCL20 low expression and low FOXP3^+^ TILs infiltration demonstrated the best OS and DFS among the 4 groups. Patients with other combinative patterns of CCL20 expression and FOXP3^+^ TILs infiltration exhibited OS and DFS in the middle of the groups (OS: χ^2^ = 25.995, *P* < .001; DFS: χ^2^ = 21.851, *P* < .001; Fig. 2E, F).

**Table 5:**
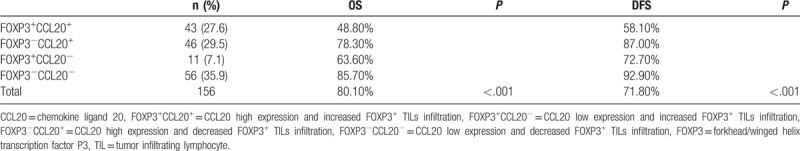
Prognostic significance of concomitant CCL20 expression and FOXP3^+^ TILs.

**Figure 3:**
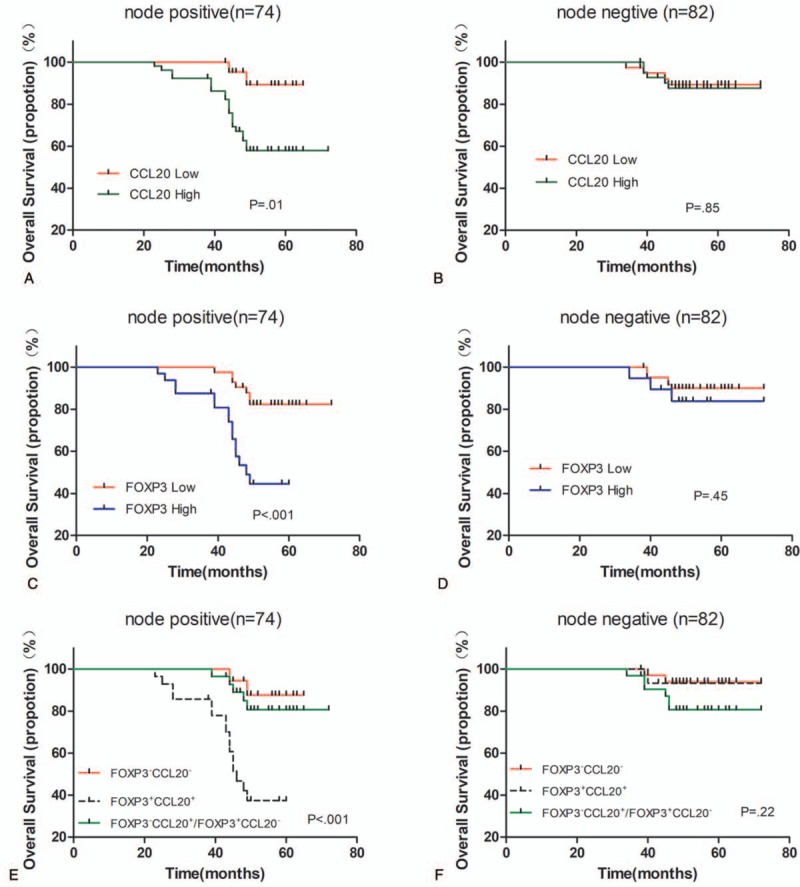
Kaplan–Meier curves for breast cancer overall survival (OS) in relation to FOXP3 and CCL20 expression in different lymph node metastasis status. A, C The OS of node-positive breast cancer patients depending on CCL20 and FOXP3 expression. B, D, The OS of node-negative breast cancer patients depending on CCL20 and FOXP3 expression. E, OS of node-positive breast cancer patients depending on the CCL20 expression combined with the tumor FOXP3^+^ TILs infiltration. F, OS of node-negative breast cancer patients depending on the CCL20 expression combined with the tumor FOXP3^+^ TILs infiltration. *P* values were calculated by the log-rank test. CCL20 = chemokine ligand 20, FOXP3^+^CCL20^+^ = CCL20 high expression and increased FOXP3^+^ TILs infiltration, FOXP3^+^CCL20^−^ = CCL20 low expression and increased FOXP3^+^ TILs infiltration, FOXP3^−^CCL20^+^ = CCL20 high expression and decreased FOXP3^+^ TILs infiltration, FOXP3^−^CCL20^−^ = CCL20 low expression and decreased FOXP3^+^ TILs infiltration, FOXP3 = forkhead/winged helix transcription factor P3, OS = overall survival.

We further evaluated the effects of FOXP3 and CCL20 expression on survival outcomes in breast cancer patients under different lymph node metastasis status. We found that in patients with axillary lymph node metastases (n = 74) that FOXP3 and CCL20 expression was correlated with survival: Patients with high CCL20 and FOXP3 expression had a shorter OS (*P* = .01; *P* < .001, Fig. 3A, C). However, in patients with no axillary lymph node metastasis (n = 82), CCL20 and FOXP3 expression did not have a significant correlation with survival (*P* = .84; *P* = .45, Fig. 3B, D). The patients with axillary lymph node metastases with the concomitant CCL20 high expression and increased FOXP3^+^ TILs infiltration had the worst OS (*P* < .001; Fig. 3E). In lymph node-negative breast cancer patients, the status of CCL20 and FOXP3 was not related to OS (*P* *=* .22; Fig. 3F). From this we can see that the effects of FOXP3 and CCL20 expression on OS were subtype specific.

### 3.5 qRT-PCR quantitation of CCL20 and FOXP3 mRNA expression in tumor tissues

**Figure 4:**
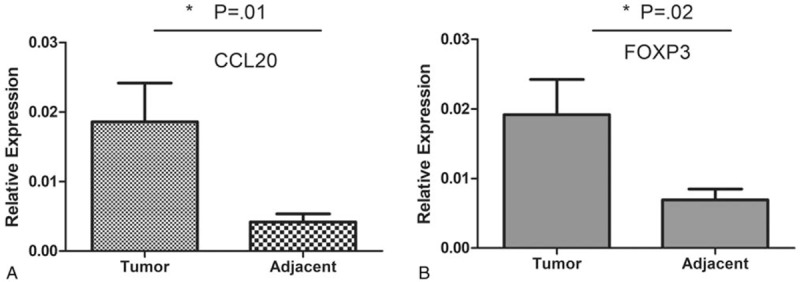
qRT-PCR analysis of FOXP3 and CCL20 expression in breast cancer tissues (n=40). A, CCL20 mRNA expression in tumor tissues was significantly greater than in NATs (*P*=.01). B, Intratumoral tissues had higher numbers of FOXP3^+^ mRNA than in NATs (*P*=.02). CCL20 = chemokine ligand 20, FOXP3 = forkhead/winged helix transcription factor P3, NAT = nontumor adjacent tissue, qRT-PCR = quantitative real time polymerase chain reaction.

In qRT-PCR, CCL20 mRNA expression in tumor tissues was significantly greater than in NATs (*P* = .01, Fig. 4A) and FOXP3 mRNA expression in tumor tissues was also significantly greater than in NATs (*P* = .02, Fig. 4B). In addition, CCL20 mRNA expression was positively correlated with FOXP3 expression in tumor tissues (r = 0.323, *P* = .04).

## 4 Discussion

The correlation between chronic inflammation and neoplastic transformation has been suggested for many years. Chronic inflammation, which produces chemokines, antigenic growth factors, and matrix-degrading enzymes, leads to a rich environment for tumor growth and invasion. CCL20/CCR6 has been detected significantly upregulated in multiple human cancers including liver, colon, pancreatic, and breast cancers, and is associated with their pathogenesis, progression, and metastasis.^[27–30]^

In this study, the immunohistochemical results showed that in breast cancer patients, high CCL20 expression and increased FOXP3^+^ TILs infiltrates were both associated with high histological grade, axillary lymph node metastases, positive HER2, and high Ki67 index. In addition, significant correlation between CCL20 expression and FOXP3^+^ TILs infiltration in breast cancer tissue was identified (rs = 0.359, *P* < .001).

We evaluated the prognostic significance of FOXP3^+^ TILs and CCL20 expression in breast cancer and conducted clinical follow-ups. Kaplan–Meier survival analysis showed that FOXP3 and CCL20 expression in tumors were strongly correlated with unfavorable prognostic factors in breast cancer patients’ cohort, the patients with the concomitant CCL20 high expression and increased FOXP3^+^ TILs infiltration showed the worst OS and DFS than those with CCL20 low expression and/or low FOXP3^+^ TILs.

Multivariate Cox regression analysis indicated that both FOXP3 and CCL20 are independent prognostic factors for OS of breast cancer patients. FOXP3 expression was also found to be a prognostic marker for DFS.

Kaplan–Meier survival analysis showed that FOXP3 expression in tumors was strongly correlated with the OS of all breast cancer patients. Results also showed that as FOXP3 staining intensity increased, the survival risk of breast cancer patients also increased. FOXP3 expression was also found to be a prognostic marker for DFS as patients with low FOXP3 expression had significantly greater DFS than patients with high FOXP3 expression.

We further evaluated the effects of FOXP3 expression on survival outcomes in breast cancer patients under different lymph node metastasis status. We found that in patients with axillary lymph node metastases, FOXP3 expression was correlated with survival: Patients with high levels of FOXP3 expression have a shorter OS. However, in patients with no axillary lymph node metastasis, FOXP3 expression did not have a significant correlation with survival. In HER-2-positive patients, patients with high FOXP3 expression had a shorter OS. However, in HER-2-negative patients, FOXP3 expression did not have a significant correlation with survival. Therefore, in the context of HER-2 and axillary lymph node-positive breast cancer, FOXP3^+^ TIL appear to serve as markers of poor prognosis. We also found that, in patients with high levels of FOXP3 expression, the high CD4 expression group had significantly worse OS than the low CD4 expression group. However, CD4 expression did not significantly correlate with survival of breast cancer patients overall. These findings also confirmed that CD4^+^FOXP3^+^ Treg can accurately represent the immunosuppressive effect of Tregs in breast cancer patients, even though FOXP3 is an important marker of Tregs.

Tregs are important immunosuppressive cells in inflammation. The transcription factor FOXP3 is the most reliable and most marker for Tregs. Studies have shown that chronic inflammation is of vital importance for the growth and metastasis of tumors because Treg infiltration not only inhibits blockade of chronic inflammation but also inhibits tumor-specific T-cell immunity and interferes with antitumor mechanisms.^[34]^

The primary function of FOXP3^+^ Tregs is to eliminate self-reactive lymphocytes, and most tumor-associated antigens are regarded as self. Therefore, they can activate Tregs and produce potential impairment of tumor immunity, thereby promoting tumor cell proliferation and metastasis.^[35–37]^

The effects of the CCL20/CCR6 axis in chemotaxis have been proven in in vitro and in vivo experiments.^[28,38]^ A study has shown that tumor cells can not only express CCR6 and CCL20 but inhibiting CCL20 expression can also reduce the proliferation, migration, and invasion of cancer cells.^[39]^ Studies found that CCR6^+^ Tregs are present in human peripheral blood and human breast cancer, nonsmall cell lung cancer, and hepatocytes.^[40–42]^ CCR6 expression can be used to identify effector/memory Tregs in mice.^[43]^

In oral squamous cell carcinoma (OSCC) cells, CCL20 and FOXP3 mRNA expression is significantly correlated. CCR6^+^ Treg cells exhibit stronger suppressive activity and display higher FOXP3 expression along with lower methylation at the Treg-specific demethylated region of the FOXP3 gene.^[44]^

The results of this study showed that FOXP3 expression is also strongly correlated with the expression of the chemokine CCL20 receptor (CCR6). qRT-PCR results also showed that the expression of FOXP3 and CCL20 mRNA in tumor tissues is greater than in NATs and CCL20 and FOXP3 mRNA expression were significantly correlated. The immunohistochemical results are consistent with qRT-PCR results. This shows that in breast cancer, CCL20 may be important in in situ recruitment or retention of FOXP3^+^ Tregs. The effects of CCL20 on Tregs may be to recruit existing FOXP3^+^ Tregs to tumor sites and inducing the expansion of retained Tregs, thereby synergistically carrying out immunosuppression.

Most studies have reported that FOXP3^+^ Tregs are a marker for poor prognosis in breast cancer.^[18,19,21]^ This is consistent with our study. However, we found that the expression of FOXP3^+^ Tregs is not correlated with survival in patients with negative axillary lymph node metastases and negative HER-2 expression. These inconsistent prognostic correlations of FOXP3^+^ Tregs reflect the complexity of immune responses in tumor tissues. In some tumors, immune infiltration occurs due to recruitment by tumor cells and promotes tumor spread. In other tumors, immune infiltration reflects the host's antitumor responses. Our study proved that FOXP3^+^ Tregs can be used as an important prognostic marker for prediction of breast cancer survival outcomes and can also be used as a therapeutic target to improve immunotherapy for breast cancer as blocking the migration or function of Tregs may aid in overcoming human cancers. In addition, CCL20/CCR6 may also be potential therapeutic targets for breast cancer.

## 5 Limitations of the study

Among patients with axillary lymph node metastases (n = 74), we found FOXP3 and CCL20 expression to be correlated with survival. Patients with high levels of CCL20 and FOXP3 expression had a shorter OS (*P* = .01; *P* < .001, Fig. 3A, C). However, we did not assess FOXP3 or CCL20 expression in metastatic axillary lymph node tissues. Moreover, the further functional linkage of FOXP3 with the expression of CD25^+^ was not addressed to compare the comorbidity of such coexpressions in determining the clinical prognosis.

## Acknowledgments

The authors thank all the patients enrolled in this study for their kind understanding and support. The authors also thank Dr Chunkai Yu, MD, Dr Hong Chang, MD, for their critical evaluations for molecular immunohistochemistry assay. Further appreciation should be addressed for pathologist Dr Yin Gao, MD, for checking the accuracy to preclude the expression of FOXP3.

## Author contributions

**Conceptualization:** Xia Zhao, Yanping Li, Jun Ren.

**Data curation:** Xia Zhao, Yanping Li, Xiaoli Wang, Shuzhen Lv.

**Formal analysis:** Xia Zhao, Jun Ren.

**Funding acquisition:** Jun Ren.

**Investigation:** Xia Zhao, Yanping Li, Shuzhen Lv.

**Methodology:** Xia Zhao, Jiangping Wu, Yanhua Yuan.

**Project administration:** Jun Ren.

**Resources:** Yanping Li.

**Software:** Xiaoli Wang.

**Supervision:** Yanping Li.

**Validation:** Xia Zhao, Xiaoli Wang.

**Writing – original draft:** Xia Zhao.

**Writing – review & editing:** Xia Zhao.
